# Occupational exposure to bioaerosols in the Norwegian salmon processing industry

**DOI:** 10.1093/annweh/wxaf038

**Published:** 2025-06-29

**Authors:** Marte Renate Thomassen, Bjørg Eli Hollund, Türküler Özgümüs, Anne Mette Madsen, Anna Beate Overn Nordhammer, Hans Thore Smedbold, Magne Bråtveit, Anje Christina Höper, Berit Bang, Miriam Grgic, Maja Karlsen Linchausen, Jorunn Kirkeleit

**Affiliations:** Department of Occupational and Environmental Medicine, University Hospital of North Norway, Hansine Hansens veg 67, 9019, Tromsø, Norway; Department of Occupational Medicine, Haukeland University Hospital, Jonas Lies vei 72, 5021, Bergen, Norway; Department of Global Public Health and Primary Care, University of Bergen, Årstadveien 17, 5009, Bergen, Norway; Department of Global Public Health and Primary Care, University of Bergen, Årstadveien 17, 5009, Bergen, Norway; National Research Centre for the Working Environment, Lersø Parkallé 105, 2100, Copenhagen, Denmark; Department of Occupational Medicine, St. Olavs Hospital, Trondheim University Hospital, Harald Hardrådes gt 14, 7030, Trondheim, Norway; Department of Occupational Medicine, St. Olavs Hospital, Trondheim University Hospital, Harald Hardrådes gt 14, 7030, Trondheim, Norway; Department of Global Public Health and Primary Care, University of Bergen, Årstadveien 17, 5009, Bergen, Norway; Department of Occupational and Environmental Medicine, University Hospital of North Norway, Hansine Hansens veg 67, 9019, Tromsø, Norway; Department of Community Medicine, UiT The Arctic University of Norway, Hansine Hansens veg 18, 9010, Tromsø, Norway; Department of Occupational and Environmental Medicine, University Hospital of North Norway, Hansine Hansens veg 67, 9019, Tromsø, Norway; Department of Medical Biology, UiT The Arctic University of Norway, Hansine Hansens veg 18, 9019, Tromsø, Norway; Department of Occupational and Environmental Medicine, University Hospital of North Norway, Hansine Hansens veg 67, 9019, Tromsø, Norway; Department of Occupational and Environmental Medicine, University Hospital of North Norway, Hansine Hansens veg 67, 9019, Tromsø, Norway; Department of Occupational Medicine, Haukeland University Hospital, Jonas Lies vei 72, 5021, Bergen, Norway; Department of Global Public Health and Primary Care, University of Bergen, Årstadveien 17, 5009, Bergen, Norway; Department of Occupational Medicine and Epidemiology, National Institute of Occupational Health, Gydas vei 8, Majorstuen, 0363, Oslo, Norway

**Keywords:** bioaerosols, endotoxin, occupational exposure, protein, salmon, seafood processing, work tasks

## Abstract

**Objectives:**

Workers in salmon processing plants are at risk of respiratory diseases. The aim of this study was to describe the Norwegian salmon processing industry in respect to production-related factors that may influence the generation of bioaerosols in the work atmosphere, and to assess salmon processing workers’ personal exposure to protein and endotoxin.

**Methods:**

The study comprised 222 workers from 9 plants. Fullshift personal exposure measurements of total protein (inhalable aerosol fraction, *n* = 380) and endotoxin (total aerosol sampler, *n* = 178) were collected on 4 consecutive workdays. Technical and process-related information was collected through plant visits and meetings with technical and production staff. Linear mixed-effect model was used, treating individuals as random effect and work area and work task within areas as fixed effects.

**Results:**

Plants differed in size, setup, processing procedures, and use of labor along the processing lines. Salmon processing overall geometric mean (GM) exposure to inhalable protein across the plants was highest in filleting area with 4.83 µg/m^3^ (geometric standard deviation [GSD] 3.16), followed by 3.91 µg/m^3^ (GSD 2.42) in slaughtering area, and 1.68 µg/m^3^ (GSD 2.40) in other areas. Endotoxin levels were generally low with the highest levels in slaughtering (GM 0.24 EU/m^3^; GSD 3.48), followed by other area (GM 0.19 EU/m^3^; GSD 4.05) and filleting (GM 0.10 EU/m^3^; GSD 2.51). The overall correlation between inhalable protein and endotoxin (total aerosol sampler) was poor (*r* = 0.13, *P* = 0.12).

**Conclusions:**

Salmon processing workers are exposed to airborne inhalable protein bioaerosols at levels similar to those measured over a decade ago, indicating that a systematic approach to reduce exposure levels is still needed. Given the known health risk, the industry and regulatory bodies need to intensify efforts to reduce exposure and protect workers’ health. The variance in exposure levels to inhalable protein across plants, areas, and tasks might form the basis for better exposure-reducing strategies.

**What Is Important With This Paper?**

This study found that the exposure of salmon processing workers to inhalable proteins is comparable to reports from a decade ago, indicating that risk of workers developing respiratory disease persists. In contrast, exposures to endotoxins were low. Exposure levels vary between work areas and work tasks, which may suggest new opportunities for exposure control strategies.

## Introduction

According to the UN Food and Agricultural Organization (FAO), an estimated 22.1 million people were employed in aquaculture in 2022, and in 2021, salmon and trout were the most traded fish products internationally (FAO 2024). Norway, the largest exporter of farmed salmon and trout globally, has had a 4-fold increase in sale of salmon in the period 1998 to 2022. According to the Norwegian directorate of fishing, 87 Norwegian processing plants had approval for slaughtering salmon in 2022. They employed more than 7000 salmon processing workers (Fiskeridirektoratet 2023). Previous research has shown that salmon processing workers are at risk of developing work-related respiratory diseases, allergy, and other hypersensitivity responses due to occupational exposure to airborne particles of biological origin (bioaerosols) generated during processing (Douglas *et al.* 1995; Shiryaeva *et al.* 2010; Fagernæs *et al.* 2024).

Salmon processing workers’ exposure to bioaerosols are likely to differ within and across various plants, departments, and work tasks (Bang *et al.* 2005; Dahlman-Hoglund *et al.* 2012; Shiryaeva *et al.* 2014). Processes with high aerosol exposure has been reported in both manual and automated operations during slaughtering and processing, as well as during specific work tasks such as cleaning surfaces and use of water hoses (Jeebhay *et al.* 2001; Dahlman-Hoglund *et al.* 2012; Shiryaeva *et al.* 2014). The bioaerosols contain bioactive agents such as high-molecular-weight allergens, enzymes, and endotoxin (Douglas *et al.* 1995; Bang *et al.* 2005; Dahlman-Hoglund *et al.* 2012; Shiryaeva *et al.* 2014).

To implement tailored exposure-reducing measures, more knowledge is needed about salmon processing workers’ bioaerosol exposure across different work areas and tasks. The aim of the present study was to describe the Norwegian salmon processing industry in respect to production-related factors that may influence the generation of bioaerosols in the work atmosphere, and to assess salmon processing workers’ personal exposure to protein and endotoxin.

## Methods

Data were collected from 9 Norwegian salmon processing plants as part of the multicenter intervention study SHInE (Effects of Interventions to Prevent Work-related Asthma, Allergy and Other Hypersensitivity Reactions in Norwegian Salmon industry workers) (Höper *et al.* 2023). While the main study comprises various data at 2 different time points, the current study includes information on exposure and exposure-related factors from baseline.

### Processing plants

Before and during data collection, walk-through surveys and meetings with management at the plants were conducted to gather information on technical and process-related factors relevant to bioaerosol exposure.

The plants operated on different schedules. Preparation of the processing line for fish intake and gill cutting typically began between 02:00 and 04:00, followed by slaughtering and filleting 1 to 4 h later. Processing of salmon normally lasts between 12 and 16 h depending on the size of the plant and the amount of fish being processed. All plants paused processing every night for cleaning the processing line. Due to study requirements described previously (Höper *et al.* 2023), samples were only collected during the day shift for about 8 h, starting with the gill cutters.

### Processing line

Salmon processing plants can be divided into 2 main processing areas: slaughtering and filleting, where the degree of handling the fish varies. Additionally, there are other areas supporting processing of salmon (eg mechanics, quality control). Lastly, there are administrative workers responsible for other tasks, mainly office work.

**Fig. 1.**
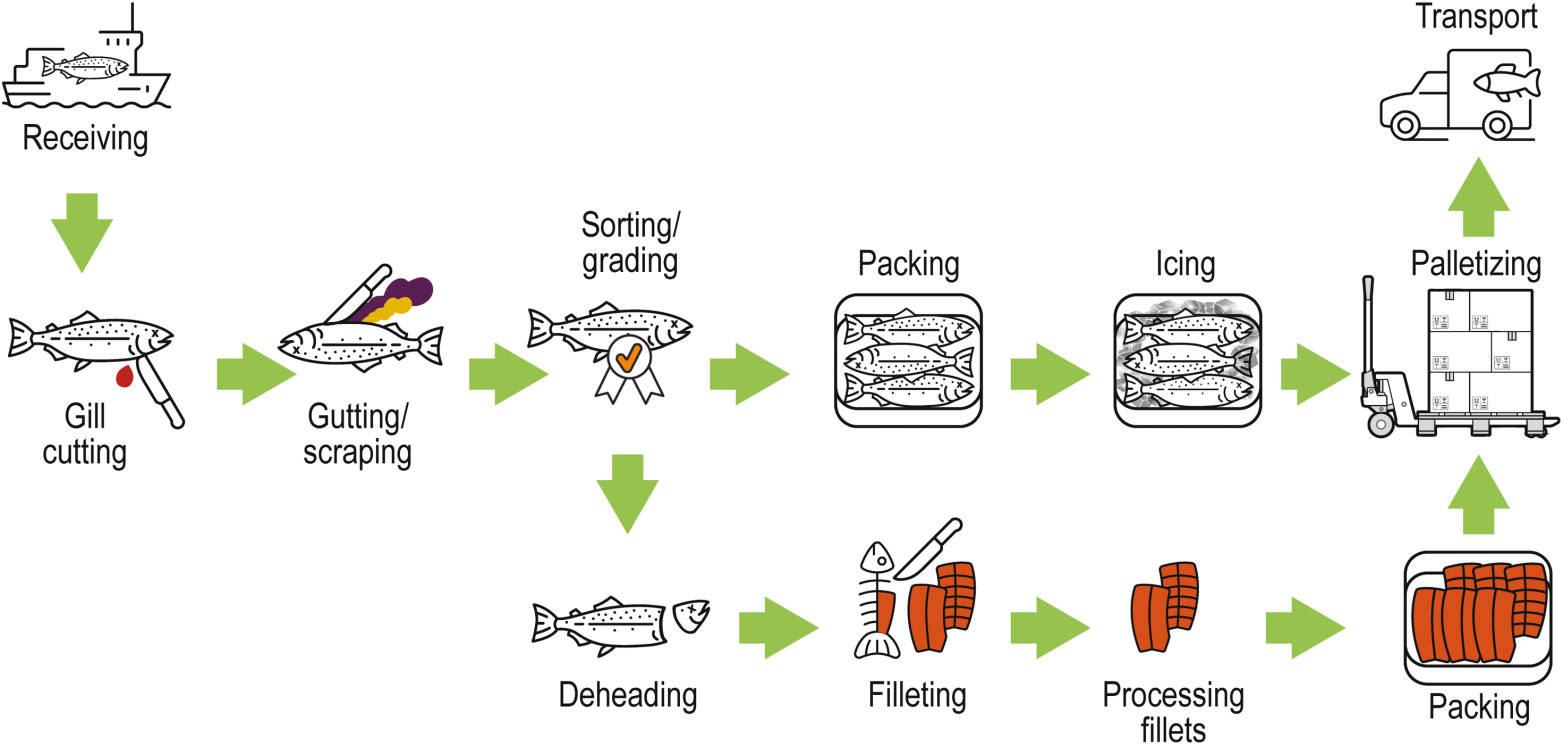
Illustration of the general workflow performed in Norwegian salmon processing plants. Illustration in collaboration with UiT, The Arctic University of Norway Graphic services.

The general layout of the processing line was similar across all 9 participating plants. Main processing tasks performed are shown in the flow chart given in Fig. 1. Work tasks commonly found in any processing plants are described in more detail in Table 1. Some of the described work tasks are not carried out in all plants, while others are carried out only when ordered by buyers. The plants varied in size, processing setup, degree of automatization, and technology. Some of the differences between the plants are highlighted in Table 2.

**Table 1.** A generic description of work tasks typically carried out in the Norwegian salmon processing industry and the main methods of production mode.

Area	Work tasks	Production mode	Description of work
Receiving area	Well boat	a/m	Transport of live fish from farming cages to processing plant. On some well boats, the fish are bled on board before delivery to the plant.
	Waiting cage	a/m	Live fish from well boats are transferred to waiting cages for de-stressing.
	Intake of fish	a	Pipes transport fish from the waiting cages or boats into the plant.
Slaughtering area			
Bleeding	Anesthetizing	a	Fish pass through an electric current or a hydraulic pin stunner. Some plants may use ice or CO_2_ in the water to anesthetize the fish.
	Gill cutting	a/m	Cutting of the gill arches/aorta with knives or other metal blades.
	Manual control of gill cutting	m	When automated cutting of gill arches, workers ensure the fish has been killed. Any live fish are killed by manual gill cutting.
Cooling tanks (Helix)		na	Fish are transported to large water tanks to bleed out and cool to desired temperature.
Degutting	Desliming	a	Water jets on conveyer belts remove the epidermal mucus layer of the fish.
	Degutting	a/m	Making an incision through the skin along the belly of the fish from the anus to the gills and remove internal organs.
	Scraping	a/m	Scraping with a vacuum-connected metal spoon inside the abdominal cavity, along the spine of the fish, for removal of organ remnants including gut, swim bladder, and kidneys.
Sorting/grading	Sorting	a/m	Fish are examined for damage, wounds, and other deformities, as well as cleanliness inside the abdominal cavity, and sorted by quality standards.
	Grading	a/m	Weighing of the fish while on conveyer belts for sorting into correct container.
Maturation		na	Vats of gutted fish are stored in cool storage areas for maturing before further processing.
Packing	Placing fish	a/m	Correct placement of fish in boxes to prevent water accumulating in fish cavities.
	Ice	a/m	Filling boxes containing fish with ice or dry ice.
	Strapping	a/m	Placing the lid, strapping the box, and labeling it before storage and transport.
Filleting area			
Descaling		a	Removal of fish scales with water jets and rotating brushes.
Deheading		a/m	Removal of the head. This task may be organized in the slaughtering area in some plants.
Filleting		a/m	Removal of the collar bone, backbone, and fins.
Trimming		a/m	Removal of the fatty belly meat as requested by the buyer.
Further processing	Removal of skin	a	Removal of skin from the fillets.
	Pinboning	a/m	Removal of bones from the fillets.
	Portioning	a/m	Sectioning the fillets into smaller pieces.
	Mincing	a	Grinding the fillets into patties for burgers or other food products.
Packing	Packing whole fillets	a/m	Placing the fillets or cuts into boxes, sealing, and labeling them.
	Vacuum packing	a/m	Placing whole fillets or smaller cuts in single vacuumed plastic containers, vacuuming, and sealing the package.
Other areas			
Quality control	Line manager	m	Ensuring the processing line runs smoothly, including participating in work tasks when necessary as well as carrying out quality control of the fish.
	Laboratory		Collecting samples from the production areas and performing microbial analyses in the lab.
Technical support		m	Maintenance and repairs on the processing line, machines, electrical system, and ventilation.
Coolers	Cold storage/freezer	a/m	Transport of boxes containing salmon by pallet trucks to storage, maintenance on the coolers and freezers.
Palletization		a/m	Transport of boxes ready for shipping on pallets or surveillance of robots palletizing. Driving pallet trucks with the ready stacked pallets to storage or on-board transport vehicles.
Ensilage		a/m	Fish residual materials can be packed and transported for food processing. Or residual materials such as internal organs, spine, and fins as well as fish no longer suitable for human food consumption can be added chemicals or packed and prepared for transport.
Transport		m	Loading pallets with boxes containing fish for transport.
Work outside the processing areas			
Office work		na	Administrative work, eg sales, human resources, work management
Cleaning^a^	Office areas Other areas	na na	Cleaning of offices, meeting rooms, cantina, wardrobes Laundry of processing workers’ clothes, gloves, and footwear

a = automatic; a/m = automatic and/or manual; m = manual; na = not applicable.

^a^Cleaning the production area is not included in the work tasks in production as the work is normally performed by specific workers only performing this work task or an external company hired in to do this work.

**Table 2.** Overview of production-related factors for the 9 different processing plants included in the study at time of inclusion.

Plant		1	2	3	4	5	6	7	8	9
Workers in production		100–200	<100	>200	>200	100–200	100–200	100–200	100–200	>200
Production volume tons/year		50–100 K	<50 K	>100 K	50–100 K	<50 K	<50 K	50–100 K	50–100 K	>100 K
Slaughtering	Gill cutting	Automated	Manual	Automated	Automated	NA	Automated	Automated	Automated	Manual
	Slaughtering machines	3 manual, 4 automated	5 manual	8 manual	8 automated	6 manual	3 manual	6 manual	6 automated	1 manual, 8 automated
	Manual gutting and scraping workstations	4 gutting, 4 scraping stations	2 gutting, 5 scraping stations	4 gutting, 4 scraping stations	6 combined gutting and scraping stations	2 combined gutting and scraping stations	3 combined gutting and scraping stations	2 gutting stations, no scraping stations	6 combined gutting and scraping stations	8 gutting, 1 scraping station
	Manual work packing^a^	Medium	High	High	Low	Low	High	Low	High	High
	Automated desliming	No	No	No	Yes	No	No	No	No	Yes
Filleting	Automated descaling	Yes	NA	Yes	Yes	Yes	Yes	Yes	No	Yes
	Deheading	Manual		Automated	Automated	Automated	Manual	Automated	Automated	Manual and automated
	Processing fillet^b^	Low		High	High	Low	Medium	High	High	High
Other factors	Number of technical staff working in plant^c^	10	7.5	13	16	12	8	16	18	33
	Workers cleaning during production using water	Spraying heads at work stations and hoses for floors	None	Spraying heads at work stations and for floors	Hoses for cleaning floors	Spraying heads at work stations and hoses for floors	Spraying heads at work stations and hoses for floors	Spraying heads for end of shift and maintenance	Spraying heads at work stations	Spraying heads at work stations, on floors to remove ice
	Truck drivers inside production area	Yes	No	No	No	Yes	No	No	No	Yes
	Handling of ensilage before transporting away from plant	Handled locally	Handled and stored locally	Handled and stored locally	Sent through vacuum pipes	Handled and stored locally	Handled and stored locally	Handled and stored locally	Handled and stored locally	Sent through vacuum pipes

K = 1000 tons; NA = not applicable.

^a^Manual work at packing; low = mainly observatory work, medium = manual placement of fish in boxes while the rest is automated, high = manual placement of fish, filling of ice, and/or manual placement of lids on boxes.

^b^Degree of handling fillets; low = manual trimming, medium = additional manual trimming, pinboning, high = use of machines for removal of skin and portioning of fillets into smaller pieces/mincing.

^c^Workers conducting daily maintenance on the production line as well as the building in general, including work on processing machines, welding, ventilation, etc.

### Study population

The study population comprised salmon processing workers from 9 salmon processing plants located along the Norwegian coastline (Höper *et al.* 2023). Participation in data collection was voluntary. Inclusion of workers was based on their planned work tasks aiming at covering different parts of the production process including workers from: (i) slaughtering, (ii) filleting, and (ii) worker groups outside, or at no specific positions within, the processing line (eg storage, maintenance, quality control, office). Other than some in the last category “Other,” the workers did not work across multiple areas. Within each area, workers would perform different work tasks (see Table 1) depending on the processing setup. Hence, the workers were allocated to the work task that they reported to have been their main work task in respect to time and potential for bioaerosol exposure on the day of sampling. While the slaughtering and filleting areas represent the processing workers, other areas represent all other workers, implying a much wider variety of work tasks and localization in the plants.

### Exposure assessment

The study period comprised 4 consecutive workdays at each of the 9 study sites (September 2021 to June 2022). In each plant, 24 workers, volunteering to carry the sampling equipment and meeting the criteria mentioned above, were asked to carry air sampling equipment for 2 work shifts each. If the workers could not carry the sampling equipment the second day, another worker would be asked to stand in. At collection, the workers were instructed by occupational hygienists on how to carry the equipment. They performed their normal work during sampling. The workers were asked to log breaks, work tasks performed during the shift, and any abnormal activity. Full shift personal exposure (approximately 8 h) to protein (inhalable aerosol fraction) and endotoxin (total aerosol sampler) was collected in the workers’ breathing zone. If their shifts exceeded 8 h, the workers would hand in the equipment in their break closest to 8 h after starting their shift. An occupational hygienist would examine the processing line during the day shift and, while doing so, ensured the equipment was carried as instructed.

#### Collection of samples.

Inhalable fraction of proteins was collected from Monday through Thursday using a conical inhalable sampler (CIS) cassette with 37 mm diameter (Casella, Bedford, UK) using Casella Apex2 sampling pumps (Casella) operated at a flow rate of 3.5 L/min. Endotoxin (total aerosol fraction) was collected on Mondays and Thursdays using SureSeal Air Monitoring Cassettes (37 mm, 3-pc, styrene, SKC Ltd., Dorset, UK) and SKC sidekick sampling pumps (SKC Ltd.) operated at a flow rate of 2.1 L/min. All samples were collected on polytetrafluoroethylene (PTFE) (1.0 µm pore, Millipore) filters on polypropylene support pads (37 mm, 1.0 µm, SKC Ltd.).

The sampler for collection of inhalable protein was carried over the workers’ right shoulder, while the sampler of endotoxin was randomly placed on the right or left side. Samplers were collected when the shift ended or after approximately 8 h. The flow rate for each sampling pump was calibrated before and measured after collection using Bios Defender 520 (SKC Ltd.). Samples were discarded if the flow rate across the sampling period was reduced by more than 20% or if the sampling pump stopped during the sampling period. In total, 6 protein and 3 endotoxin samples were discarded. The sampling followed the same protocol across all 9 locations.

After sampling the protein, filters were stored at −18 °C and shipped to The University Hospital of North Norway on dry ice for analysis. The endotoxin filters were stored at room temperature until they were shipped to The National Research Centre of the Working Environment in Denmark for analysis.

### Analysis of samples

#### Total protein (inhalable aerosol fraction).

Samples collected on filters were extracted in 0.7 mL extraction buffer (0.5% Tween-20, 1× PBS), for 4 h at +4°C by orbital rotation (20 rpm; IKA Loopster Basic, Germany). Following the extraction, the suspension was centrifuged (1200 × *g*; MicroCL 21, Thermo Scientific, USA) for 10 min to remove particulates. The supernatant was used for protein analysis.

Protein amount was determined using BCA assay (QuantiPro BCA Assay Kit, Sigma-Aldrich, USA) in 96-well plates. Each sample (100 µL) was mixed with 100 µL BCA assay buffer, prepared according to QuantiPro BCA Assay Kit protocol (Sigma-Aldrich protocol) and incubated at 37 °C for 2 h. Each sample was run in duplicates. Post incubation, the absorbance was measured using a plate reader (Multiskam FC, Thermo Scientific) at 540 nm wavelength. A bovine serum albumin (BSA, 1 mg/mL, Sigma-Aldrich) standard was used in dilution range 0 to 30 µg/mL. The concentration of extracted protein was calculated using linear equation based on BSA standard curve using GraphPad Prism (GraphPad Prism, version 9 for Windows, GraphPad Software, San Diego, CA, USA). Samples above the range of the standard curve were diluted 10× with extraction buffer and reanalyzed. The lower level of protein detection in filter extracts was 1.545 µg/mL as determined by using the standard deviation of 11 independent analyses of a blank filter extract multiplied by 3.

#### Endotoxin (total aerosol sampler).

The endotoxin was extracted in 4.5 mL sterile solution (0.05% Tween-20) by orbital shaking (300 rpm) at room temperature for 60 min, and subsequently, the suspension was centrifuged (1000 × *g*) for 15 min to remove particulates. The samples were analyzed using the recombinant Factor C (rFC) assay (Lonza Inc., Switzerland). Sample extracts were prepared using sterile, certified pyrogen-free water with 0.05% Tween-20. All extracts from the first 2 plants were diluted 2, 10, 20, and 50 times to determine relevant dilution levels. All samples were below the detection level if diluted more than 10 times, and subsequent samples were therefore diluted 1.5, 2, or 10 times to obtain quantifiable level. Diluted samples were added to a 96-well plate, followed by 100 μL of a mixture of the fluorogenic substrate, buffer, and rFC enzyme (Lonza, Inc., Switzerland). Plates were incubated at 37 °C for 1 h and read in a fluorescence microplate reader (Pyrowave XM Fluorescence Reader, Lonza, Switzerland) at excitation and emission wavelengths of 380 and 440 nm. Background fluorescence was subtracted, and fluorescence intensity change in logarithmic scale was plotted against log endotoxin concentrations of 0.005, 0.05, 0.5, and 5 EU/mL. Limit of detection (LOD) for endotoxin (total aerosol sampler) was 0.005 to 0.0075 EU/m^3^, dependent on sampling time. Field blanks were collected on all days of sampling and used during analyses. All field blanks were below the detection levels.

To be able to compare endotoxin exposure levels obtained by the rFC assay with already published data, endotoxin was also quantified using the Limulus Amoebocyte Lysate assay (LAL assay). For this, 41 randomly collected samples from plants 1, 3, 4, and 9 were used. Endotoxin was measured using the LAL assay (Kinetic-QCL 50-650U kit, Lonza, Walkersville, MD, USA) with a standard curve from 0.05 to 50 EU/mL as described previously (Douwes *et al.* 1995). The endotoxin concentrations using the LAL assay were significantly higher than concentrations measured using the rFC assay (geometric means [GMs] = 0.23 vs 0.053 EU/m^3^, *n* = 41, *P* < 0.0001, pairwise comparison). The correlation between endotoxin concentrations obtained using the 2 methods was *r* = 0.76 (*P* < 0.0001). The ratio of concentrations measured using the LAL assay vs the rFC was 4.3. For any comparisons of endotoxin levels with publications using LAL assay, the results of this study were multiplied with 4.3.

### Statistical analyses

The data are presented as arithmetic means (AMs) with 95% confidence intervals (CIs) and GMs with geometric standard deviations (GSDs) (Tables 3 and 4). As the exposure data showed right-skewed distribution, the measurements were log-transformed. Unless otherwise specified, results are presented as GMs.

**Table 3.** Salmon processing workers’ exposure to inhalable fraction protein (µg/m^3^) in 9 Norwegian salmon processing plants (2021–2022). The estimates are adjusted for repeated measurements using a linear mixed model. The results are presented as all samples combined (overall) for each of the 3 areas (slaughtering, filleting, and other), and for separate work tasks within each area across all 9 plants based on what was reported by the workers to be the main task the day of sampling.

Exposure variables	Workers (*n*)	*N*	AM	95% CI	GM	GSD
Departments (overall, 9 plants)						
Slaughtering	85	144	6.08	4.88–7.58	3.91	2.42
Gill cutting	19	32	5.64	3.99–7.96	4.00	1.99
Gutting/scraping	44	74	7.42	5.15–10.69	4.28	3.01
Sorting/grader	12	18	3.54	2.75–4.55	3.14	1.27
Packing slaughtery	10	20	4.62	2.66–8.03	2.94	2.47
Filleting	55	92	8.58	6.19–11.89	4.83	3.16
Deheading/descaling	11	18	19.02	4.03–89.81	5.31	12.84
Processing fillets^a^	35	57	8.03	5.44–11.86	4.94	2.64
Packing fillets	9	17	6.18	2.86–13.34	3.79	2.65
Other	79	134	2.60	2.11–3.21	1.68	2.40
Operational office^b^	15	27	3.56	2.32–5.47	2.50	2.03
Office (unexposed)^c^	13	21	1.75	0.94–3.25	1.06	2.72
Maintenance	11	21	2.00	1.13–3.53	1.31	2.32
Quality/operational control	7	15	2.57	1.41–4.7	1.83	1.99
Truck/storage	28	44	2.59	1.76–3.81	1.74	2.21
Ensilage	3	3	3.93	2.24–6.87	3.77	1.08
Unspecified	2	3	1.36	0.44–4.22	1.15	1.40

^a^Trimming, removal of skin, pinboning, portioning, and mincing.

^b^Operational offices were technical/automation staff surveying the process on computers, often with windows overlooking the processing areas and could sometimes go into the processing areas.

^c^Office (unexposed) were administrative staff and workers with similar tasks that did not directly interact with the processing line.

**Table 4.** Salmon processing workers’ exposure level to endotoxin (EU/m^3^) (total aerosol sampler) in the 9 Norwegian salmon processing plants. The results are presented as all samples combined for each of the 3 work areas (slaughtering, filleting, and other), and for work tasks within each area across all 9 plants based on what was reported by the workers to be the main task the day of sampling. Endotoxin was determined using the rFC assay.

Exposure variables	Workers (*n*)	Measurements (*N*)	AM	95% CI	GM	GSD
Departments (overall, 9 plants)						
Slaughtering	48	84	0.52	0.35–0.77	0.24	3.48
Gill cutting	13	20	0.17	0.09–0.32	0.10	2.75
Gutting/scraping	24	45	0.75	0.47–1.2	0.38	3.23
Sorting/grader	7	10	0.52	0.18–1.46	0.24	3.43
Packing slaughtery	4	9	0.33	0.29–0.52	0.17	3.11
Filleting	23	32	0.15	0.12–0.92	0.10	2.51
Deheading/descaling	5	6	0.33	0.1–0.23	0.20	2.77
Processing fillets^a^	14	19	0.12	0.11–1.03	0.08	2.52
Packing fillets	4	7	0.10	0.07–0.21	0.09	1.77
Other	20	42	0.51	0.27–0.96	0.19	4.05
Operational office^b^	5	11	0.62	0.2–1.91	0.28	3.46
Maintenance	3	6	0.62	0.08–4.72	0.18	4.88
Quality/operational control	1	2	0.45	0.07–2.76	0.29	2.53
Truck/storage/fridge	11	23	0.48	0.18–1.31	0.15	4.52

NA = not applicable.

^a^Trimming, removal of skin, pinboning, portioning, and mincing.

^b^Operational offices were technical/automation staff surveying the process on computers, often with windows overlooking the processing areas and could sometimes go into the processing areas.

In total, 25 samples (6.5%) of protein and 12 samples of endotoxin were below LOD. According to the method by van Buuren (2007), multiple imputation of protein concentrations below LOD was performed using the following variables: measurement time (min), protein filter number, a dummy variable showing whether protein concentration was below LOD, work task, work area, and company. Total variation was calculated by applying Rubin’s rules (Rubin 1987). Mice package and “leftcenslognorm” method was used.

When estimating workers’ exposure for protein and endotoxin, a linear mixed-effect model was used to include the effect of repeated individual measurements (random effects) including work area and work task within areas as fixed effects.

Univariate analysis of variance (ANOVA) was used to test differences in exposure levels between the exposure categories and plants (multiple groups), and Scheffe’s post hoc test of difference between these groups. The Students *t*-test was used for comparison between 2 groups. Correlations between concentrations of protein and endotoxin overall and within the 3 work areas (slaughtering, filleting, and other) were described by Pearson’s correlation coefficient (*r*).

Analyses were performed using R version 4.3.2, with R packages; here (v1.0.1), tidyverse (v2.0.0), flextable (v0.9.1), ggtext (v0.1.2), lme4 (v1.1.35-1), mice (v3.16.0), qpcomp (v2.15.2), and merDeriv (v0.2.4).

## Results

A total of 222 workers including stand-ins participated in the exposure assessment, comprising 380 measurements of inhalable protein and 178 measurements of endotoxin (total aerosol sampler). The average sampling time for protein and endotoxin were 478 min and 486 min (range 75 to 720 min), respectively.

### Processing plants

The 9 processing plants differed in size, setup, processing procedures, and use of automation or manual labor on the processing lines. The central areas in the processing line are presented in Table 2. Administrative work tasks are not included as these are not suspected to cause differences in bioaerosol production between the plants.

### Exposure to inhalable protein

The exposure levels of inhalable protein were stratified by the 3 main areas: slaughtering, filleting, and other with their respective work tasks are presented in Table 3.

**Fig. 2.**
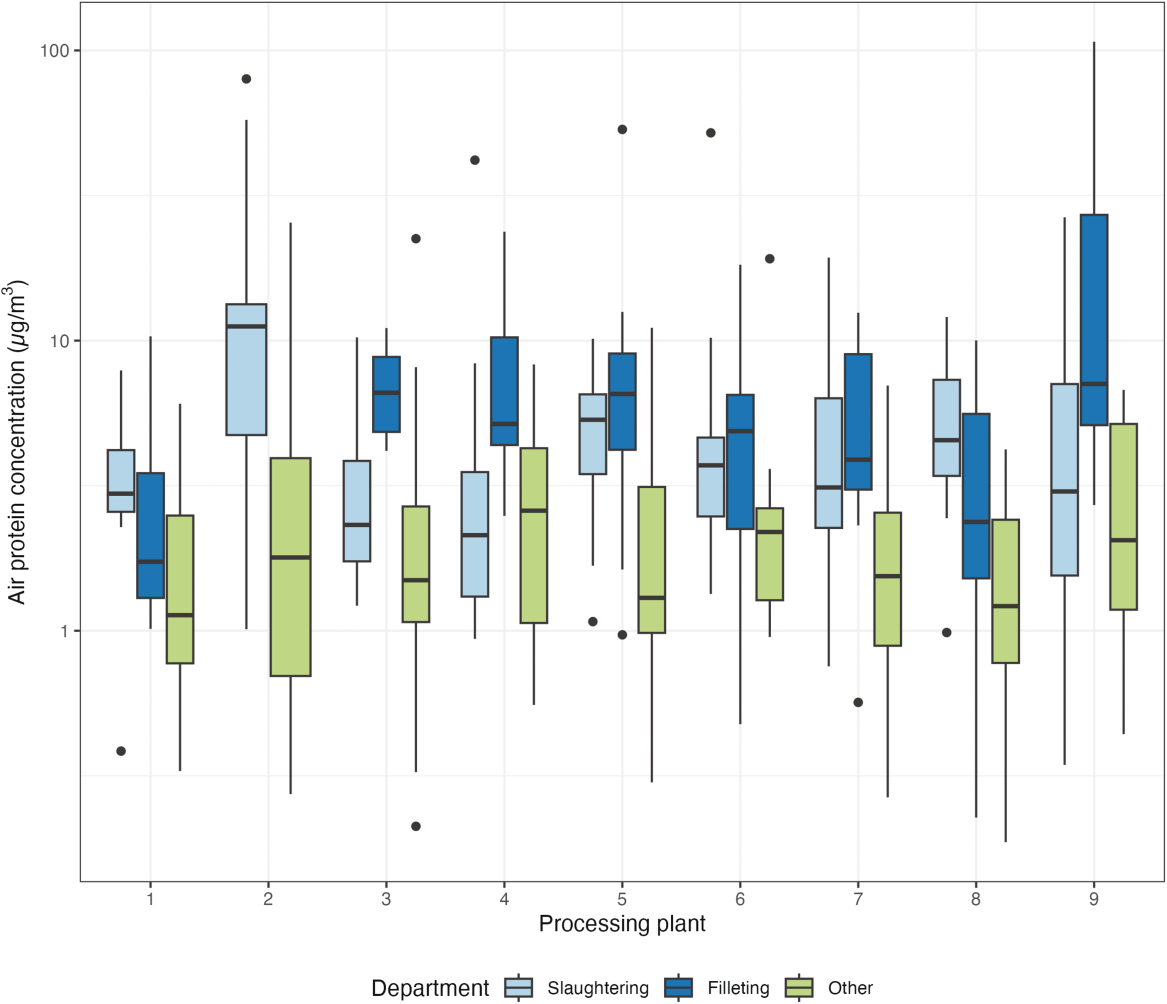
Box plot of personal exposure to inhalable protein (µg/m^3^, on a log scale) in 9 salmon processing plants at slaughtering, filleting, and other areas. Plant 2 did not have a filleting area. The box plot shows median values with its quartiles, 5 and 95 percentiles (lines), and outliers (dots).

Exposure to protein differed significantly between the areas (ANOVA, *P* < 0.001), ascribed to the difference between the lower exposed workers allocated to work in other areas and the areas slaughtering (*P* < 0.001) and filleting (*P* < 0.001) (Fig. 2). The difference between slaughtering and filleting were statistically significant in plants 3 (*P* ≤ 0.001), 4 (*P* < 0.001), and 9 (*P* = 0.024), where the exposure was highest in the filleting departments.

### Exposure to endotoxin

Exposure to airborne endotoxin (total aerosol sampler) measured using the rFC assay stratified by 3 main areas: slaughtering, filleting, and other, and their respective work tasks are presented in Table 4. No samples of endotoxin were taken in administrative areas since these samples were collected on Tuesdays and Wednesdays when there was no endotoxin sampling. Levels of endotoxin exposure were generally low compared to both the suggested occupational exposure limit (OEL) and other studies in the seafood industry. The slaughtering department had the highest (0.24 EU/m^3^) mean levels followed by the other department not directly handling the product (0.19 EU/m^3^), and filleting had the lowest exposure (0.10 EU/m^3^).

### Correlations between protein and endotoxin

The overall correlation coefficient between concentrations of endotoxin and protein was poor (*r* = 0.13, *P* = 0.12). When stratifying on work areas, the correlation was strengthened for filleting (*r* = 0.49, *P* = 0.004) but not for slaughtering (*r* = 0.16, *P* = 0.14) or other areas (*r* = 0.13, *P* = 0.45).

## Discussion

In this study, comprising 9 salmon processing plants and 222 participating workers, we found that salmon processing workers’ exposure to inhalable protein and endotoxin varies between plants as well as between departments and work tasks within the plants. Comparisons of bioaerosol levels between studies are hampered by the lack of OELs as well as standardized methods for collection and analyses of measurements (Douwes *et al.* 1995; Eduard *et al.* 2012). The correlation between personal exposure to inhalable protein and endotoxin was poor, suggesting that interventions aiming at reducing protein may not affect endotoxin and vice versa.

The plants in this study differed in size, production volume, machinery, and processing setup. Previously, factors such as layout, ventilation, processing size, and equipment have been described as covariates contributing to the “plant effect” on measured exposure levels (Jeebhay *et al.* 2005). In the present study, we report on variation in protein exposure both between departments within the pant and across different plants. To identify key determinants of exposure, further analyses using appropriate statistical methods is needed.

### Exposure to inhalable protein

The personal exposure to inhalable protein for slaughters and filleters in the present study were 3.9172 and 4.83 µg/m^3^, respectively. There is a lack of data on salmon processing workers exposure to bioaerosols. However, one study in the Norwegian salmon processing industry from 2007 to 2008 reported a GM exposure to protein (total aerosol fraction) of 2.7 µg/m^3^ (range 0.76 to 12.6) across the work week for workers in the slaughtering and filleting areas combined (Shiryaeva et al. 2014). Considering the different sampling methodologies used in the 2 studies, the exposure levels are likely comparable since the total dust sampler used in the study by Shiryaeva has been reported to underestimate the exposure level compared to the sampler used for the inhalable aerosol fraction (Notø *et al*. 2016). This indicates no significant reduction in bioaerosol exposure over the years despite known health risks. Increased production volumes with less manual and more automated processing, including the introduction of aerosol-generating machineries, have probably contributed to increased aerosol exposure in many plants. Hence, despite established knowledge about salmon processing workers’ excess risk of respiratory diseases due to occupational exposure to bioaerosols, the industry has not been able to implement exposure-reducing measures that have resulted in noticeable diminutions in the exposure levels of protein over the past 15 yr.

Salmon processing workers’ exposure to inhalable protein was higher in filleting and slaughtering areas compared to the other area which included work tasks away from the processing line. Slaughtering and filleting areas did not differ significantly from each other in exposure to inhalable protein except for in 3 plants. In plant 3, 4, and 9, the protein exposure was highest in the filleting area (Fig. 2). All 3 plants had a rather high number of processing workers (more than 200 employees), a production volume in the higher end, automated deheading, high degree of manual work in the filleting area, and high degree of processing filets (Table 2). With all this work on the fish as well as the large scale of processing in the filleting departments, it is likely that the proteins would be aerosolized during handling of the exposed meat and cause higher levels of exposure.

Although previously reported exposure levels to bioaerosols generated during processing of salmon are scarce, there are some reports on personal exposure to protein among workers in other seafood processing industries which show large variations in exposure levels. Protein (thoracic fraction) was measured in 2 South African factories processing anchovy, pilchard, and rock lobster. The overall personal exposure not stratified by worker groups was 0.73 µg/m^3^ (GM), while the highest exposure measurement of 11.5 µg/m^3^ was associated with technical maintenance/workshop (Jeebhay *et al.* 2005). Exposure to inhalable protein among seafood processing workers in Greenland in 2016 varied between types of seafood being processed: fish (6.1 µg/m^3^, max. 25.7), snow crab (20.3 µg/m^3^, max. 61.3), and shrimp (50.0 µg/m^3^, max. 1.285) (Laustsen *et al.* 2022). Exposure to protein during processing of crab was reported to be 5.1 µg/m^3^ (range 1.1 to 48.0) in king crab and 11.9 µg/m^3^ (range 3.4 to 47.2) in edible crab (Thomassen *et al.* 2016). Our findings of mean levels up to 5.31 µg/m^3^ in deheading/descaling and an overall mean exposure of 4.83 and 3.91 µg/m^3^ in filleting and slaughtering departments, respectively, indicate that the exposure in other seafood processing industries generally is higher. But the distributions of exposures are generally large in this industry.

### Exposure to endotoxin

In the present study, processing workers’ exposure to endotoxin (total aerosol sampler) was low with most AMs and GMs being below 2% of the available Dutch OEL of 90 EU/m^3^ (Health Council of the Netherlands 2010), also after using the conversion factor of 4.3 from rFC to Limulus. The endotoxin exposure was higher in slaughtering compared to filleting. In the slaughtering area, large volumes of seawater and other debris are pumped in from the well boats or waiting cages, and large water-filled helixes where the fish bleed out (Table 1). Additionally, large amounts of water are used during gutting. All these processes are likely to produce bioaerosols and droplets of water containing biological material that deposit on surfaces where bacteria can grow until the plants are cleaned at night. Air samples taken during this study at the same time as the bioaerosol samples were collected show that there are several gram-negative bacteria present as bioaerosols in the production area at low concentrations (Madsen *et al.* 2024). The highest concentrations of the gram-negative bacteria were found in the early stages of the processing chain, the same areas as the highest levels of endotoxin were found. These bacteria are likely a source of endotoxin found in the bioaerosol measurements.

Exposure levels to endotoxin in the present study were in the lower end of previous reports from the salmon processing industry (Bang *et al.* 2005; Dahlman-Hoglund *et al.* 2012; Shiryaeva *et al.* 2014). Two studies in Norwegian salmon processing plants reported that slaughters had an AM and median endotoxin exposure of 7.9 EU/m^3^ (SD 10.5) and 3.3 EU/m^3^ (range 0.9 to 36.0), respectively (Bang *et al.* 2005), while salmon processing workers overall had a mean exposure of 1.52 EU/m^3^ (range 0.30 to 29.0) (Shiryaeva *et al.* 2014). Among Swedish salmon processing workers, the reported endotoxin concentrations ranged from 1.6 to 7.1 EU/m^3^ depending on the work task (Dahlman-Hoglund *et al.* 2012).

In general, the reported endotoxin levels in the salmon industry have been considerably lower than in other types of seafood industry, including processing of sardines (Jeebhay *et al.* 2005), cod (Bang *et al.* 2005; Laustsen *et al.* 2022), herring (Bang *et al.* 2005), crustaceans (Ortega *et al.* 2001; Thomassen *et al.* 2016; Laustsen *et al.* 2022), and fish meal (Jeebhay *et al*. 2004) reporting mean concentrations ranging between 0.14 and 136 EU/m^3^. Previous studies have shown that fish pathogens have the capacity to form biofilm on stainless steel (Vidal *et al.* 2020) and that some bacteria can survive and grow at low temperatures and high salinity (Sudagidan *et al.* 2021). Bacteria found by Madsen *et al.* (2024) have also been found in seawater (Daae *et al.* 2024) and in the fish industry (Møretrø and Langsrud 2017). The low levels of endotoxin may be due to the industry’s stringent hygienic standards for cleaning and disinfection of processing equipment and areas as shown in another study from a fish processing plant (Guðbjörnsdóttir *et al*. 2005). However, even though the exposure levels in our study were low, endotoxin was present. Studies in airway cell models indicate a synergistic effect on inflammation when low concentrations of endotoxin are combined with the enzyme trypsin (Bhagwat *et al.* 2016). Salmon-derived trypsin is previously shown to be present in salmon industry bioaerosol samples (Bang *et al.* 2018). Exposure to trypsin in the SHInE study population will be elaborated in a later article.

The correlation between inhalable protein and endotoxin (total aerosol sample) was poor. This may be due to the differences in sources for the components. The source of endotoxin is gram-negative bacteria, while the source for protein is materials of biological origin in general. This includes the fish itself in addition to plants or animal debris in seawater. In a separate publication, we have shown that gram-negative bacteria are most abundant during gill cutting and gutting, both in the slaughtering department (Madsen *et al.* 2024). Exposure to protein tended to be higher in the filleting departments (Fig. 2). At the current level of knowledge, a conversion factor translating available measurements of protein to endotoxin levels, or vice versa, is not applicable in this industry. Further, exposure-reducing interventions aimed at reducing exposure to protein will not necessarily affect endotoxin levels and vice versa.

### Bioaerosol exposure in areas outside the processing line

The main sources of bioaerosols in salmon processing plants are likely handling of fish through cutting meat with knives, scrapers, and pressurized water, as well as using water to clean the fish, remove mucus and organ remnants, and spray from conveyer belts. However, the exposure levels reported in the present study indicate that workers not directly involved in processing of the salmon (eg technical support, quality control, ensilage) have a potential for exposure to both protein and endotoxin comparable to the levels reported for processing workers (slaughtering and filleting areas). Similar findings have been reported with work tasks such as cleaning of the processing line (Jeebhay *et al.* 2001; Jeebhay *et al*. 2004). This indicates that both maintenance tasks and the background level of aerosols in the areas might contribute to the exposure.

Ensilage work is a task with direct contact with fish residual material or fish that is not primarily processed further for human consumption, and that is either stored in silos locally or transported away. Workers’ reporting having ensilage as their main work task had a mean exposure to inhalable protein of 3.8 µg/m^3^. However, this estimate was based on only 3 measurements from one plant (plant 5). Ensilage is a heterogenous task that needs to be better characterized in respect to bioaerosol exposure.

Combining maintenance and cleaning during production resulted in the highest measured endotoxin concentrations in our study. Cleaning with use of water hoses during production was associated with increased exposure to allergens, proteins, and endotoxins in salmon processing plants (Shiryaeva *et al.* 2014). Workers involved in maintenance often rotate between different areas and work in areas where equipment is left due to malfunctions. The equipment may be left to be handled by maintenance workers and so are not cleaned, leaving time for bacteria to grow. Since bacteria is the direct source of endotoxin, maintenance workers may be more likely to be exposed to endotoxin when they handle this equipment than workers along the processing line handling fresh salmon.

### Strengths and limitation

The present study is, to our knowledge, the largest and most comprehensive exposure assessment of salmon processing workers’ exposure to protein and endotoxin comprising personal samples (380 protein and 178 endotoxin samples) collected from 222 workers.

The inclusion of a large variety of salmon processing plants in respect to locations, size, and processing procedures makes the present study representative for the salmon processing industry in Norway. Out of 87 registered salmon processing plants operating in Norway in 2021, 9 (10.3%) were included in the study. All plants asked to participate were willing to do so, reducing the potential for selection bias. Workers carrying the sampling equipment were selected based on their current work assignments and only excluded if they declined to participate or knew they could not be present during the whole sampling period. There is no reason to believe the participants work was systematically different from other workers doing the same work tasks.

Comparison of exposure levels of protein and endotoxin within and across seafood industries, as well as evaluation of risk of health effects due to exposure levels, are challenging due to the lack of standardized methods for collection and analysis of the samples and lack of relevant OELs (Douwes *et al.* 1995; Eduard *et al.* 2012). Previous studies in the seafood industry have mainly measured protein in the total aerosol fraction. In general, inhalable samplers are preferred for bioaerosols since they in most cases comprise particles of varying sizes (Eduard *et al.* 2012), and the inhalable fraction provides a more accurate representation of the particles and droplets entering the workers respiratory system. Inhalable fraction could also be useful for the needed establishment of OELs for bioaerosols of marine origin. The SureSeal Air Monitoring Cassettes (37 mm closed-face cassettes [CFCs]) used for endotoxin collection are known to underestimate the inhalable aerosol levels, especially for large particles sizes (Harper and Muller 2002; Görner *et al.* 2010; Hanlon *et al*. 2021). For instance, in agricultural settings, the sampling efficiency of CFC/The SureSeal Air Monitoring Cassettes varies from 49% to 67% compared to IOM inhalable sampler (Hanlon *et al*. 2021). However, it is difficult to say anything certain about the aerosols we collected since we do not know the particle size distributions.

Workers in the Norwegian salmon processing industry are a mixture of Norwegian-born workers, immigrant workers residing in Norway, and foreign workers hired from temporary staff recruitment agencies. Although written information about the study and questionnaires were provided in 6 languages, the instructions for carrying and registering sampling equipment was only given in Norwegian and English. Language barriers might have affected the communication with the participating workers regarding the oral instructions on how they should carry the sampling equipment and what information was needed in the work log. It might also have affected the field workers’ ability to perceive possible sources of errors or deviations from the protocol. To prevent this, all sampling equipment was prepared and packed by the researchers, and the equipment was placed on the workers to ensure their proper placement. Observations were also made throughout the shift by occupational hygienists to make sure the sampling progressed as planned.

A shortcoming of the study is that we do not have sampling from some specific locations since workers carrying sampling equipment alternated between different work tasks during one shift. We also lack detailed information on specific work tasks such as use of water hoses. This type of data would allow linkage of the concentration of inhalable protein to specific areas, work tasks, use of water, and workers’ behavior that would allow for identification of the most important bioaerosol-generating procedures and tailored implementation of specific bioaerosol-reducing measures along the processing line. This study could also have benefited from more detailed analyses of other factors expected to contribute to variations in exposure levels such as building-related factors, arrangements of the processing line, machinery, and ventilation.

## Conclusions

Salmon processing workers are exposed to protein bioaerosols at levels similar to those measured over a decade ago, indicating that measures to reduce these levels have not been sufficient. Exposure to airborne endotoxin seems to be lower than reported in previous studies. More knowledge is needed about the effectiveness and feasibility of various types of exposure-reducing measures. Given the known risk of respiratory diseases from the different bioaerosol components, it is crucial for the industry and regulatory bodies to intensify efforts to reduce exposure to protect workers’ health.

## Data Availability

The participant-level data set will not be available for public access owing to the GDPR. Metadata and statistical syntaxes beyond those reported in this publication will be available upon reasonable request.

## References

[CIT0002] Bang B , et al 2005. Exposure and airway effects of seafood industry workers in northern Norway. J Occup Environ Med. 47:482–492. https://doi.org/10.1097/01.jom.0000161732.96555.2b 15891527

[CIT0001] Bang BE , Malla N, Bhagwat SS, Aasmoe L, Winberg JO. 2018. A sensitive assay for proteases in bioaerosol samples: characterization and quantification of airborne proteases in salmon industry work environments’. Ann Work Expo Health. 62:942–952. https://doi.org/10.1093/annweh/wxy050 29947734

[CIT0003] Bhagwat SS , Larsen AK, Seternes OM, Bang BE. 2016. Mixed exposure to bacterial lipopolysaccharide and seafood proteases augments inflammatory signalling in an airway epithelial cell model (A549). Toxicol Ind Health. 32:1866–1874. https://doi.org/10.1177/0748233715590914 26149191

[CIT0007] Daae HL , et al 2024. A cross-sectional study on occupational exposure to microorganisms, endotoxin, hydrogen sulfide, and dust during work at drilling waste treatment plants. Ann Work Expo Health. 68:58–77. https://doi.org/10.1093/annweh/wxad069 37995292 PMC10773208

[CIT0004] Dahlman-Hoglund A , Renstrom A, Larsson PH, Elsayed S, Andersson E. 2012. Salmon allergen exposure, occupational asthma, and respiratory symptoms among salmon processing workers. Am J Ind Med. 55:624–630. https://doi.org/10.1002/ajim.22067 22576678

[CIT0005] Douglas JD , et al 1995. Occupational asthma caused by automated salmon processing. Lancet. 346:737–740. https://doi.org/10.1016/s0140-6736(95)91505-2 7658875

[CIT0006] Douwes J , Versloot P, Hollander A, Heederik D, Doekes G. 1995. Influence of various dust sampling and extraction methods on the measurement of airborne endotoxin. Appl Environ Microbiol. 61:1763–1769. https://doi.org/10.1128/aem.61.5.1763-1769.1995 7646014 PMC167439

[CIT0008] Eduard W , Heederik D, Duchaine C, Green BJ. 2012. Bioaerosol exposure assessment in the workplace: the past, present and recent advances. J Environ Monit. 14:334–339. https://doi.org/10.1039/c2em10717a 22267210 PMC4687010

[CIT0009] Fagernæs CF , et al 2024. Occupational asthma in the salmon processing industry: a case series. Occup Environ Med. 81:400–406. https://doi.org/10.1136/oemed-2024-109564 39137970

[CIT0011] Fauske M and Fiskeridirektoratet. 2023. Nøkkeltall fra norsk havbruksnæring 2022. p. 28. https://www.fiskeridir.no/statistikk-tall-og-analyse/data-og-statistikk-om-akvakultur/statistiske-publikasjon-innen-akvakultur/_/attachment/inline/3655df0f-5734-4255-a181-54d6c66853f9:1e9471f22426f3e0729f482b1e9057eb6ffb66ac/nokkeltall-havbruk-2022.pdf

[CIT0010] Food and Agriculture Organization of the United Nations. 2024. *The State of World Fisheries and Aquaculture 2024—Blue Transformation in Action*. Rome.

[CIT0013] Görner P , Simon X, Wrobel R, Kauffer E, Witschger O. 2010. Laboratory study of selected personal inhalable aerosol samplers. Ann Occup Hyg. 54:165–187. https://doi.org/10.1093/annhyg/mep079 20147627

[CIT0012] Guðbjörnsdóttir B , Hjorleifur E, Gudjon T. 2005. Microbial adhesion to processing lines for fish fillets and cooked shrimp: influence of stainless steel surface finish and presence of gram-negative bacteria on the attachment of *Listeria monocytogenes*. Food Technol Biotechnol. 43:55–61. https://www.ftb.com.hr/images/pdfarticles/2005/January-March/43-55.pdf

[CIT0014] Hanlon J , Galea KS, Verpaele S. 2021. Review of workplace based aerosol sampler comparison studies, 2004-2020. Int J Environ Res Public Health. 18:6819. https://doi.org/10.3390/ijerph18136819 34202035 PMC8296900

[CIT0015] Harper M , Muller BS. 2002. An evaluation of total and inhalable samplers for the collection of wood dust in three wood products industries. J Environ Monit. 4:648–656. https://doi.org/10.1039/b202857n 12400909

[CIT0016] Health Council of the Netherlands, The Hauge. 2010. *Endotoxins—Health-Based Recommended Occupational Exposure Limit, Publoication Nr. 2010/04OSH*. https://www.healthcouncil.nl/documents/advisory-reports/2010/07/15/endotoxins-health-based-recommended-occupational-exposure-limit

[CIT0017] Höper AC , et al 2023. Effects of Interventions to Prevent Work-Related Asthma, Allergy, and Other Hypersensitivity Reactions in Norwegian Salmon Industry Workers (SHInE): protocol for a pragmatic allocated intervention trial and related substudies. JMIR Res Protoc. 12:e48790. https://doi.org/10.2196/48790 37467018 PMC10398556

[CIT0020] Jeebhay MF , et al 2005. Environmental exposure characterization of fish processing workers. Ann Occup Hyg. 49:423–437. https://doi.org/10.1093/annhyg/meh113 15705596

[CIT0018] Jeebhay MF , Robins TG, Lehrer SB, Lopata AL. 2001. Occupational seafood allergy: a review. Occup Environ Med. 58:553–562. https://doi.org/10.1136/oem.58.9.553 11511741 PMC1740192

[CIT0019] Jeebhay MF , Robins TG, Lopata AL. 2004. World at work: fish processing workers. Occup Environ Med. 61:471–474. https://doi.org/10.1136/oem.2002.001099 15090672 PMC1740773

[CIT0021] Laustsen BH , Ebbehøj NE, Sigsgaard T, Rasmussen K, Bønløkke JH. 2022. Work environment, occupational diseases and accidents among seafood industry workers in Greenland. Dan Med J. 69:A05210470. https://content.ugeskriftet.dk/sites/default/files/scientific_article_files/2022-01/a05210470_web.pdf 35088702

[CIT0022] Madsen AM , et al 2024. Airborne bacterial and fungal species in workstations of salmon processing plants. Sci Total Environ. 951:175471. https://doi.org/10.1016/j.scitotenv.2024.175471 39137839

[CIT0023] Møretrø T , Langsrud S. 2017. Residential bacteria on surfaces in the food industry and their implications for food safety and quality. Compr Rev Food Sci Food Saf. 16:1022–1041. https://doi.org/10.1111/1541-4337.12283 33371605

[CIT0024] Notø HP , Nordby KC, Eduard W. 2016. Relationships between personal measurements of ‘total’ dust, respirable, thoracic, and inhalable aerosol fractions in the cement production industry. Ann Occup Hyg. 60:453–466. https://doi.org/10.1093/annhyg/mev093 26755796

[CIT0025] Ortega HG , et al 2001. Respiratory symptoms among crab processing workers in Alaska: epidemiological and environmental assessment. Am J Ind Med. 39:598–607. https://doi.org/10.1002/ajim.1059 11385644

[CIT0026] Rubin, DB. 1987. Multiple imputation for nonresponse in surveys. John Wiley & Sons, Inc.

[CIT0028] Shiryaeva O , et al 2014. Respiratory effects of bioaerosols: exposure-response study among salmon-processing workers. Am J Ind Med. 57:276–285. https://doi.org/10.1002/ajim.22281 24310925

[CIT0027] Shiryaeva O , Aasmoe L, Straume B, Bang BE. 2010. Respiratory impairment in Norwegian salmon industry workers: a cross-sectional study. J Occup Environ Med. 52:1167–1172. https://doi.org/10.1097/JOM.0b013e3181fc5e35 21124247

[CIT0029] Sudagidan M , et al 2021. Bacterial surface, biofilm and virulence properties of *Listeria monocytogenes* strains isolated from smoked salmon and fish food contact surfaces. Food Bioscience 41:101021. https://doi.org/10.1016/j.fbio.2021.101021

[CIT0030] Thomassen MR , et al 2016. Occupational exposure to bioaerosols in Norwegian crab processing plants. Ann Occup Hyg. 60:781–794. https://doi.org/10.1093/annhyg/mew030 27235847

[CIT0031] van Buuren S. 2007. Multiple imputation of discrete and continuous data by fully conditional specification. Stat Methods Med Res. 16:219–242. https://doi.org/10.1177/0962280206074463 17621469

[CIT0032] Vidal JM , et al 2020. Formation of biofilms of the salmon pathogen *Flavobacterium psychrophilum* in different surfaces using the CDC biofilm reactor. Aquaculture. 514:734459. https://doi.org/10.1016/j.aquaculture.2019.734459

